# Advancing metabolic engineering of *Yarrowia lipolytica* using the CRISPR/Cas system

**DOI:** 10.1007/s00253-018-9366-x

**Published:** 2018-09-21

**Authors:** Tian-Qiong Shi, He Huang, Eduard J. Kerkhoven, Xiao-Jun Ji

**Affiliations:** 1 College of Biotechnology and Pharmaceutical Engineering, Nanjing Tech University, No. 30 South Puzhu Road, Nanjing, 211816 People’s Republic of China; 2 School of Pharmaceutical Sciences, Nanjing Tech University, No. 30 South Puzhu Road, Nanjing, 211816 People’s Republic of China; 3 State Key Laboratory of Materials-Oriented Chemical Engineering, Nanjing Tech University, No. 5 Xinmofan Road, Nanjing, 210009 People’s Republic of China; 4 Jiangsu National Synergetic Innovation Center for Advanced Materials (SICAM), No. 5 Xinmofan Road, Nanjing, 210009 People’s Republic of China; 5 Systems and Synthetic Biology, Department of Biology and Biological Engineering, Chalmers University of Technology, Gothenburg, Sweden

**Keywords:** CRISPR/Cas, Metabolic engineering, *Yarrowia lipolytica*, Genome editing

## Abstract

The oleaginous yeast *Yarrowia lipolytica* is widely used for the production of both bulk and fine chemicals, including organic acids, fatty acid-derived biofuels and chemicals, polyunsaturated fatty acids, single-cell proteins, terpenoids, and other valuable products. Consequently, it is becoming increasingly popular for metabolic engineering applications. Multiple gene manipulation tools including URA blast, Cre/LoxP, and transcription activator-like effector nucleases (TALENs) have been developed for metabolic engineering in *Y. lipolytica*. However, the low efficiency and time-consuming procedures involved in these methods hamper further research. The emergence of the CRISPR/Cas system offers a potential solution for these problems due to its high efficiency, ease of operation, and time savings, which can significantly accelerate the genomic engineering of *Y. lipolytica*. In this review, we summarize the research progress on the development of CRISPR/Cas systems for *Y. lipolytica*, including Cas9 proteins and sgRNA expression strategies, as well as gene knock-out/knock-in and repression/activation applications. Finally, the most promising and tantalizing future prospects in this area are highlighted.

## Introduction

*Yarrowia lipolytica* is a well-known non-conventional yeast, which is generally recognized as safe (Liu et al. 2015). Due to its strong lipogenesis capability and high protein expression levels, *Y. lipolytica* is widely researched for the production of both bulk and fine chemicals, including organic acids, fatty acid-derived biofuels and chemicals, polyunsaturated fatty acids, single-cell proteins, terpenoids, and other valuable products (Rymowicz et al. 2010; Cui et al. 2011; Yin et al. 2012; Xue et al. 2013; Kamzolova et al. 2014; Blazeck et al. 2015; Sun et al. 2016; Liu et al. 2017a, b; Gao et al. 2017). Meanwhile, a large range of substrates can be effectively utilized by *Y. lipolytica*, including not only glucose and glycerol but also xylose, cellobiose, and other industrial wastes, which has made it into a hot topic of recent biorefinery research (Ledesma-Amaro and Nicaud 2016; Zeng et al. 2018). Metabolic engineering is a rapidly developing field that purposely uses genetic recombination technologies to modify cellular metabolic pathways, change cell characteristics, and combines with other technologies such as biochemical engineering to construct new metabolic pathways for the synthesis of specific products (Stephanopoulos 2012; Nielsen and Keasling 2016; Chen et al. 2017). For instance, the overexpression of the endogenous acetyl-CoA carboxylase (ACC1) and diacylglycerol acyltransferase (DGA1) genes in *Y. lipolytica* increased the lipid content to 41.4%, a 4.7-fold improvement over the parental strain (Tai and Stephanopoulos 2013). Subsequently, the aldehyde dehydrogenase gene was introduced to improve the strain’s resistance to oxidative stress, after which the lipid content reached up to 90% (Xu et al. 2017). Metabolic engineering was also employed to produce other products in *Y. lipolytica* in recent years with good results, which attracted increasing attention to the metabolic engineering research of this yeast (Yin et al. 2012; Beopoulos et al. 2014; Rutter et al. 2015; Blazeck et al. 2015; Kildegaard et al. 2017; Liu et al. 2017a, b). With the increasing number of metabolic engineering studies in *Y. lipolytica*, various genetic engineering tools have been developed to meet the demands (Hussain et al. 2016). These tools include URA blast, TRP1 blast, Cre/LoxP systems for recycling selection markers, and transcription activator-like effector nucleases (TALENs) for gene knock-out and knock-in (Cheon et al. 2003; Fickers et al. 2003; Rigouin et al. 2017; Gao et al. 2017). While these tools have been established in *Y. lipolytica*, they suffer from various limitations and remain not fully conducive to efficient, high-throughput genetic engineering.

**Fig. 1:**
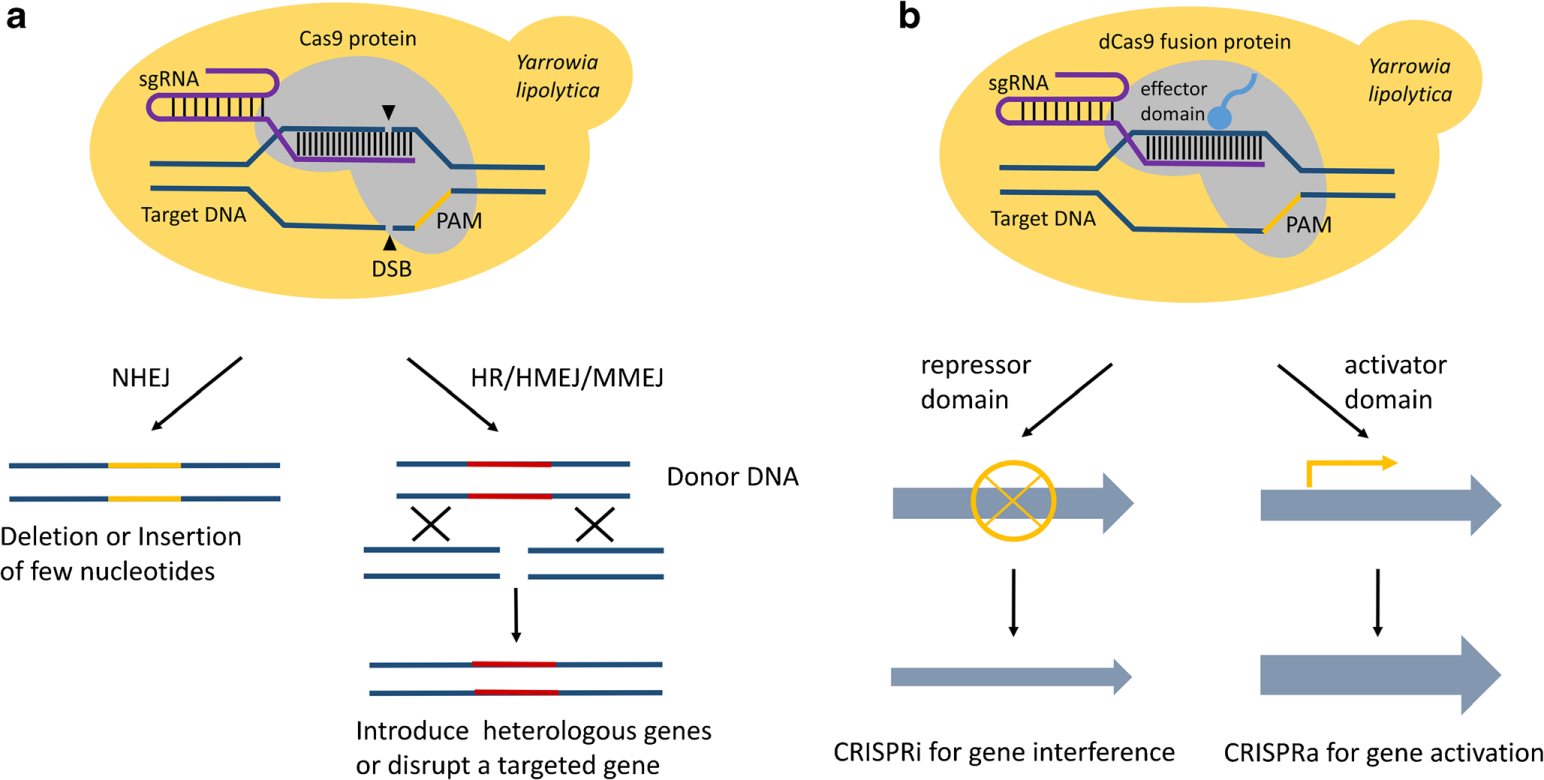
The CRISPR/Cas genome editing platform for *Yarrowia lipolytica*. **a** CRISPR/Cas9 method for gene knock-out/knock-in. When the sgRNA recognizes the targeted sequence, which is located before a protospacer adjacent motif (PAM) site, the Cas9 protein will catalyze the formation of a double-strand break (DSB) in the targeted DNA. In order to repair the genome, two kinds of repair mechanisms can be used. The non-homologous end-joining (NHEJ) repair mechanism, which is dominant in *Y. lipolytica*, can quickly repair the genome at the expense of the deletion or insertion of a few nucleotides, which can lead to the frameshift mutations in the targeted gene. Additionally, in the presence of a homologous sequence, cells can use the donor DNA to introduce nested heterologous genes or disrupt a targeted gene by homologous recombination (HR), homology-mediated end-joining (HMEJ), and microhomology-mediated end-joining (MMEJ) repair mechanisms. **b** CRISPRi and CRISPRa methods for gene interference and activation, respectively. A catalytically deactivated Cas9 (dCas9), which has no cleavage activity, can be fused with different effector domains to control gene expression. When the targeted region is recognized, the dCas9 fusion protein with the transcriptional repressor domain binds the DNA to repress gene expression. Similarly, the fusion protein of dCas9 and a transcriptional activator domain binds to targeted regions to improve the gene expression level

The CRISPR/Cas system, which emerged at an opportune time, to some extent solves the traditional problems. The CRISPR/Cas system consists of mainly two components, a Cas9 protein and the corresponding sgRNA (Shi et al. 2017). As shown in Fig. 1, CRISPR/Cas systems based on different types of Cas proteins can be classified into three groups—knock-out/in-oriented CRISPR/Cas9, CRISPR interference (CRISPRi), and CRISPR activation (CRISPRa) (Sharma et al. 2017). When the sgRNA recognizes the targeted sequence, the Cas9 protein catalyzes a double-strand break (DSB) in the targeted DNA, which induces either random deletion and insertion or the introduction of heterologous genes through partially complementary donor DNA (O’Connell et al. 2014; Ran et al. 2013). CRISPRi is used for gene repression via a catalytically deactivated Cas9 (dCas9), which has no cleavage activity, but can nevertheless bind the DNA and repress the expression of the gene targeted by the gRNA (Larson et al. 2013). In order to enhance the repression activity, transcriptional repressors, such as Krüppel associated box (KRAB) domain, is usually expressed as a fusion with the Cas9 protein (Zhang et al. 2018). Similarly, CRISPRa was developed for targeted gene activation by fusing dCas9 to transcriptional activators that bind promoters of targeted genes and improve gene expression levels (Simeonov et al. 2017). These technologies offer important solutions, including multi-gene targeting and marker-free integration, which promote the development of metabolic engineering in *Y. lipolytica*.

In this review, we summarize the expression strategies and recent applications of the CRISPR/Cas system in *Y. lipolytica*, followed by a brief discussion of future prospects of this system. We hope to provide a practical reference for genome editing in *Y. lipolytica*.

## Development of a CRISPR/Cas9 system for *Y. lipolytica*

### Cas9/dCas9 expression strategies

As the first CRISPR research in *Y. lipolytica* by Schwartz et al. (2016a), both expression of the active Cas9 protein in the CRISPR/Cas9 system and dCas9 in CRISPRi and CRISPRa systems after that has been engineered using a strong constitutive promoter as well as a SV40 nuclear localization signal (Gao et al. 2016; Schwartz et al. 2017; Schwartz et al. 2018; Schwartz and Wheeldon 2018; Holkenbrink et al. 2018; Zhang et al. 2018). There are two general strategies for the expression of Cas9/dCas9—one based on plasmids and the other on chromosomal integration. In the plasmid-based setup, Cas9/dCas9 can be cloned into an autonomously replicating plasmid (ARP) for recycling of marker genes, or a non-ARP for transient expression, both of which showed a high editing efficiency in *Y. lipolytica* (Schwartz et al. 2016a). In addition, Holkenbrink et al. (2018) established an EasyCloneYALI genetic toolbox in which Cas9 is integrated into the genome for easier transformation protocols. With this system, highly efficient genome editing only requires an sgRNA expression cassette, a strategy that is also of interest for CRISPRi and CRISPRa systems.

### sgRNA expression strategies

#### Promoter engineering to improve the genome editing efficiency

Efficient genome editing mainly depends on the level of sgRNA transcription. Therefore, many studies have focused on promoter engineering in recent years. Gao et al. (2016) and Wong et al. (2017) used a polymerase II promoter (Pol II) to transcribe sgRNAs in *Y. lipolytica*. In this system, sgRNAs were flanked by hammerhead (HH) and hepatitis delta virus (HDV) ribozymes. Due to the strength of Pol II and self-processed RNA cleavage, sgRNAs can be successfully released. Similar research was also previously carried out by Schwartz et al. (2016a). However, the efficiency was quite low, and in order to further improve the editing efficiency, Schwartz et al. (2016a) adopted a synthetic RNA polymerase III promoter (Pol III) strategy in which differently designed Pol III promoters were individually and combinatorically used to optimize the knockout efficiency. The SCR1-tRNA^Gly^ hybrid Pol III achieved an efficiency of nearly 100% after 4 days of outgrowth, and was consequently frequently utilized to express sgRNAs in later studies. In very recent research, Morse et al. (2018) established a T7 polymerase-based CRISPR/Cas system in different yeasts, including *Y. lipolytica*. Here, the sgRNAs were expressed from a T7 promoter which was transcribed by a heterologous mutant T7 polymerase, and the genome editing efficiency reached nearly 60%. The establishment of promoter engineering strategies has laid the foundation for further development of the CRISPR/Cas system in *Y. lipolytica*.

#### Multiplex sgRNA expression strategy

Multiplex sgRNA expression strategies have been widely applied to the CRISPR/Cas system in *Y. lipolytica*. Gao et al. (2016) investigated the efficiency of simultaneous single, double, and triple gene disruption in *Y. lipolytica*. The final result showed that the frequency of single-disruption events approached 100%, while for double disruption, it was nearly 36%, and reached 19% for triple disruption. Holkenbrink et al. (2018) established a multi-sgRNA expression system, in which multiplex sgRNAs that target different genes can be constructed rapidly through the BioBricks assembly. Additionally, a multiplex sgRNA expression strategy has been used for CRISPRi and CRISPRa (Zhang et al. 2018; Schwartz et al. 2017); however, in these cases, the multiplex sgRNAs were usually designed to target a single desired gene rather than multiplex different genes. The reasoning for this is that when the targeting is biased towards a single gene, the efficiency can be lower, and multi-gene targeting has a better efficacy for gene interference and activation. These examples demonstrate that multiplex sgRNA expression plays an important role in the CRISPR/Cas system.

## CRISPRS/Cas system for knock-out/knock-in and repression or activation of genes

### Gene knock-out or knock-in

In the field of metabolic engineering, a highly efficient homologous recombination (HR) system of microbes is essential for gene knock-out/knock-in, which in turn is a prerequisite for investigating the functions of the targeted genes. However, the system is in direct competition with the stronger non-homologous end-joining (NHEJ) mechanism in most organisms including *Y. lipolytica* (Ueno et al. 2007; Decottignies 2007). Consequently, a high HR efficiency usually requires homologous arms with a length of more than 1 kb. In order to enhance the HR efficiency, chemical and biological approaches for inactivation of the NHEJ repair pathway in *Y. lipolytica* were adopted. Verbeke et al. (2013) identified the ku70 and ku80 genes in *Y. lipolytica*, which play a role in the NHEJ repair mechanism after the formation of DSB. While the disruption of ku80 did not affect the HR efficiency, it was significantly improved by ku70 disruption. In the corresponding knockout strain, the efficiency of HR mediated by only 50-bp homologous arms can be increased to 43%. Interestingly, Kretzschmar et al. (2013) proved that ku80 disruption can also increase the HR efficiency to 80% with long homologous arms 1 kb in length. Moreover, they observed the highest HR efficiency up to 85% with ku70 disruption. In addition, based on the ku70 disruption, Jang et al. (2018) added hydroxyurea into the medium to synchronize the cell cycle to the S-phase, which has been proved to induce the HR in *Y. lipolytica* (Tsakraklides et al. 2015). The experiment demonstrated that 50-bp homologous arms can yield an HR efficiency of 46% and 100-bp homologous arms can reach up to 100%. Although these strategies have been developed to improve HR efficiency, it is quite difficult to knock out multiple genes simultaneously even in an NHEJ-knockout strain. Moreover, the use of multiple selection markers is not conducive to further metabolic engineering and industrial utilization of the resulting strains (Wagner and Alper 2016). Fortunately, the emergence of the CRISPR/Cas system offers the possibility to solve these problems.

CRISPR/Cas9 system for *Y. lipolytica* was first established by Schwartz et al. (2016a). In their research, more attention was paid to finding the best Pol III promoter to improve the genome editing efficiency. The final result indicated that a combination of SCR1 and tRNA Pol III promoters was the best choice. Based on these results, a standardized markerless gene integration tool for pathway engineering was subsequently established by Schwartz et al. (2016b). By knocking out 17 genes using an autonomously replicating CRISPR/Cas9 plasmid as well as the repair fragment, five loci which offer efficient gene integration were identified. These repair fragments as well as the CRISPR/Cas9 plasmids together comprise a standardized tool that allows efficient genome editing in any of the five loci. In order to verify the practical applicability of this tool, the multigene pathway for lycopene biosynthesis was subsequently integrated into the *Y. lipolytica* genome. Importantly, repair fragments were designed to easily insert any targeted genes, and these plasmids can be removed in a day, which has the potential to significantly accelerate the construction of any metabolic pathway in *Y. lipolytica*.

Comparable to the above approach, Holkenbrink et al. (2018) established a CRISPR/Cas9-based toolbox for engineering *Y. lipolytica*. In the system, Cas9 and sgRNA were separately expressed from two different plasmids, and the Cas9 protein was subsequently integrated into the genome. A total of 11 loci which did not affect cell growth were selected to design the sgRNAs and repair fragment. Both marker-mediated integration and CRISPR/Cas9-based marker-free genome editing had a high efficiency. Additionally, the use of this toolbox for multiplex gene knockouts was tested. For single gene disruption, the efficiency was above 80%, and it varied from 6 to 66% for double gene disruption. However, for triple gene disruption, no successful transformants were found. To simplify the plasmid construction process, 90-bp double-stranded oligonucleotides were used as the template to repair the DSB by HR, and the editing efficiency reached 100%, which further demonstrated the validity of this toolbox.

Interestingly, Gao et al. (2018) recently established a dual-sgRNA-mediated gene knockout and integration strategy for *Y. lipolytica*. By designing paired sgRNAs for single genes, both non-coding and coding regions of the targeted gene could be cleaved precisely. The result was further confirmed by knocking out six genes. Moreover, based on a new homology-mediated end-joining (HMEJ) strategy, which was recently established in animal embryo and tissue cells (Xuan et al. 2017; Yao et al. 2018), researchers also applied this HMEJ strategy to *Y. lipolytica*. Strikingly, the efficiency was twice as high as that of HR.

Taken together, fast developments of the CRISPR/Cas9 system provide a great deal of convenience for metabolic engineering in *Y. lipolytica*. Both marker-free integration and multi-gene editing are powerful tools to overcome traditional shortcomings in HR systems, facilitating further metabolic engineering of this yeast.

### Sequence-specific repression or activation of genes

In the CRISPR/Cas9 system, the original purpose of the Cas9 protein was to bind the DNA and cleave the targeted gene sequence. However, it was found that a dCas9 variant which has no cleavage activity can also specifically bind the targeted DNA (Ma et al. 2016). Importantly, dCas9 can be fused to transcriptional repressors and activators to further repress or activate gene expression. Subsequently, CRISPRi and CRISPRa manipulations have been quickly applied to many different organisms, including *Y. lipolytica*.

Schwartz et al. (2017) were the first to establish the CRISPRi system in *Y. lipolytica*. The purpose was to repress NHEJ to enhance HR efficiency. In the verification experiments, eight of nine target genes were efficiently repressed. In order to further improve the HR efficiency, a multiplex sgRNA expression strategy as well as a dCas9 fusion protein with the Mxi1 repressor was adopted to repress the ku70 and ku80 genes. The subsequent rate of HR was nearly 90%. Additionally, a microhomology-mediated end-joining (MMEJ) mechanism, which is independent of the NHEJ mechanism, was found in *Y. lipolytica*. Homology regions of only 8 bp can be used to repair the genome with the MMEJ after DSB formation. Subsequently, Zhang et al. (2018) used the four different repression proteins dCpf1, dCas9, dCas9-KRAB, and dCpf1-KRAB, to rapidly develop the CRISPRi system in *Y. lipolytica*. Notably, the researchers found that there was no explicit relation between target sites and repression efficiency. Therefore, multiplex sgRNAs were simultaneously expressed to improve the system’s efficiency, and rates of gene repression as high as 85% and 92% were achieved using dCpf1 and dCas9, respectively. Furthermore, the possibility of double and triple gene interference using the CRISPRi system was explored in this research. The final results showed that the combined repression events successfully occurred in *Y. lipolytica*, which demonstrated that CRISPRi was indeed a powerful tool for metabolic engineering of *Y. lipolytica*.

In addition to the gene interference system, the dCas9 protein has also been fused to transcription activators to activate the target genes, which is known as CRISPRa. Based on the previously established CRISPRi technology, Schwartz and Wheeldon (2018) rapidly developed CRISPRa manipulation in *Y. lipolytica*. Considering that transcription activators have an enormous influence on the activation efficiency, researchers firstly compared four different activators and found that the synthetic tripartite activator VPR yielded the highest activation. After that, the dCas9-VPR fusion protein was used to activate two β-glucosidase genes—BGL1 and BGL2—which enabled *Y. lipolytica* to grow on cellobiose robustly. By designing multiplex sgRNAs targeting the promoters of the two β-glucosidase genes, researchers eventually found that sgRNAs near to the core promoter region could greatly increase the activation degree. The expression level of BGL1 increased 112-fold, while that of BGL2 increased 43-fold. Moreover, the activation of both genes simultaneously also yielded a high efficiency.

In summary, dCas9-mediated gene repression and activation is playing increasingly more important roles. Consequently, more silent regions in the genome of *Y. lipolytica* can be activated to explore their encoded functions. Furthermore, the correlation between gene expression and cellular phenotypes can be understood in significantly more detail, which will further deepen metabolic engineering research in *Y. lipolytica*.

## Applications of the CRISPR/Cas system in *Y. lipolytica*

As shown in Table 1, the CRISPR/Cas system has been quickly applied in metabolic engineering of *Y. lipolytica* following its introduction.

**Table 1:** Recent applications of the CRISPR/Cas system in *Yarrowia lipolytica*

sgRNA expression strategy	Cas9/dCas9 expression strategy	Application	Editing efficiency	References
SCR1-tRNA^Gly^ ; HH-HDV	Plasmid-based	Knockout studies of XDH and XKS	–	Rodriguez et al. 2016
SCR1-tRNA^Gly^	Plasmid-based	CRISPR/Cas9 tool for targeted, markerless gene integration	~ 50%	Schwartz et al. 2016b
SCR1-tRNA^Gly^	Plasmid-based	Disrupting TRP1	–	Wagner et al. 2018
SCR1-tRNA^Gly^ ; dual sgRNA cleavage	Plasmid-based	A dual-cleavage strategy for gene integration	14.3–32.6%	Gao et al. 2018
tRNA promoter; Biobrick assembly; multiplex sgRNA target	Genomic integration	CRISPR/Cas9 tool for marker-free gene integration	90%	Holkenbrink et al. 2018
HH-HDV; multiplex sgRNA target	Plasmid-based	CRISPR/Cas9 tool for gene knockout	28–98%	Gao et al. 2016
SCR1-tRNA^Gly^	Plasmid-based	Disrupting TRP1	–	Markham et al. 2018
SCR1-tRNA^Gly^	Plasmid-based	CRISPR/Cas9 tool	~ 90%	Schwartz et al. 2016a
SCR1-tRNA^Gly^	Plasmid-based	Knocking out glycogen synthesis	–	Bhutada et al. 2017
T7 promoter	Plasmid-based	Knocking out the CAN1 gene	60%	Morse et al. 2018
SCR1-tRNA^Gly^ ; multiplex sgRNA target	Plasmid-based	Enhancing HR	90%	Schwartz et al. 2017
SCR1-tRNA^Gly^ ; Golden-Brick assembly; multiplex sgRNA target	Plasmid-based dCas9 and dCas9-KRAB	CRISPRi tool for gene repression	92%	Zhang et al. 2018
SCR1-tRNA^Gly^ ; multiplex sgRNA target	Plasmid-based dCas9-VPR fusion protein	CRISPRa system for activation of cryptic sugar metabolism	–	Schwartz and Wheeldon 2018
HH-HDV	Plasmid-based	YaliBricks-based CRISPR/Cas9 tool	12.5%	Wong et al. 2017

–, not stated

Rodriguez et al. (2016) used CRISPR/Cas9 to knock-out the xylulose kinase and xylitol dehydrogenase genes in *Y. lipolytica* in the xylose metabolic pathway. The knockout strains demonstrated that both genes are essential for xylitol/xylose metabolism. Based on these result as well as further study in *Escherichia coli*, researchers engineered xylose utilization in *Y. lipolytica*, which enabled it to grow on xylose robustly. Bhutada et al. (2017) used the CRISPR/Cas9 system to knock out the glycogen synthase gene in *Y. lipolytica* because they found it was too challenging to knock out this gene using HR in a triacylglycerol (TAG) synthesis-deficient strain. The final result showed that glycogen synthesis played a competing role in the TAG accumulation process and the deletion of this gene improved the lipid content by 60%. Markham et al. (2018) engineered *Y. lipolytica* to produce triacetic acid lactone. In this research, the TRP1 gene was disrupted by CRISPR/Cas9 to introduce an available selection marker. Similar to this research, Wagner et al. (2018) also knocked out TRP1 with CRISPR/Cas9 in order to establish a piggyBac transposon system in *Y. lipolytica*.

Compared to the gene knock-out/knock-in-oriented CRISPR/Cas9 method, both CRISPRi and CRISPRa methods are still in their infancy. Although Schwartz et al. (2017) and Zhang et al. (2018) individually established the CRISPRi system, and Schwartz and Wheeldon (2018) subsequently established the CRISPRa system, there are few applications of these two methods in *Y. lipolytica*. However, we believe that the need to engineer *Y. lipolytica* for the tailored production of specific chemicals will greatly expand the use of CRISPRi and CRISPRa systems in metabolic engineering research of this organism in the near future.

## Conclusions and perspectives

Compared to the widely used yeast model organism *Saccharomyces cerevisiae*, the non-conventional yeast *Y. lipolytica* has a stronger lipogenesis ability. Therefore, *Y. lipolytica* has been increasingly explored for the production of lipid-related products via metabolic engineering. The adaptation of genetic tools from *S. cerevisiae* to *Y. lipolytica* would enable more rapid and convenient strain engineering and facilitate reaching the full potential of *Y. lipolytica*. The emergence and application of the CRISPR/Cas system undoubtedly accelerates the rate of metabolic engineering for *Y. lipolytica* strain improvement. Although the CRISPR/Cas system has been firmly established in this yeast, there still is much room for further improvement. For instance, problems related to multi-gene editing efficiency, more precise site-directed mutagenesis in the genome as well as high-throughput screening technology after genome editing need to be addressed. However, we believe that these problems can be solved and that increasing numbers of applications of CRIPSR/Cas in *Y. lipolytica* will quickly come available in the near future.
